# Acute portal vein thrombosis precipitated by indomethacin in a HCV-positive elderly patient

**DOI:** 10.1186/1471-2318-12-69

**Published:** 2012-11-13

**Authors:** Stefania Mantarro, Marco Tuccori, Giuseppe Pasqualetti, Sara Tognini, Sabrina Montagnani, Fabio Monzani, Corrado Blandizzi

**Affiliations:** 1 Tuscan Regional Centre for Pharmacovigilance, Interdepartmental Centre for Research in Clinical Pharmacology and Experimental Therapeutics, University of Pisa, Via Roma 55, Pisa, 56126, Italy; 2 Tuscan Regional Centre for Pharmacovigilance, Unit of Adverse Drug Reaction Monitoring, University Hospital of Pisa, Via Roma 55, Pisa, 56126, Italy; 3 Geriatric Unit, Department of Clinical and Experimental Medicine, University Hospital of Pisa, Via Roma 67, Pisa, 56126, Italy; 4 Tuscan Regional Centre for Pharmacovigilance Division of Pharmacology, Department of Clinical and Experimental Medicine, University of Pisa, Via Roma 55, Pisa, 56126, Italy

**Keywords:** Portal vein thrombosis, Indomethacin, Chronic HCV infection, Case report

## Abstract

**Background:**

An increased risk of venous thromboembolism has been reported in patients treated with non-steroidal anti-inflammatory drugs (NSAIDs). We describe a case of acute portal vein thrombosis (PVT) in a hepatitis C virus (HCV)-positive elderly patient following administration of indomethacin.

**Case presentation:**

A 79-year-old HCV-positive man was hospitalized for severe abdominal pain, nausea and vomiting, 15 days after starting indomethacin for back pain. Clinical signs and imaging evaluations disclosed a picture of PVT. Indomethacin was discontinued, and the patient was started on fondaparinux and antithrombin. He was discharged 15 days later due to improvement of his clinical conditions. Thirty days later, a follow-up ultrasound did not show appreciable signs of PVT. The time elapsing between the start of analgesic therapy and PVT onset suggests a role of indomethacin as the triggering agent. Indomethacin could have precipitated PVT by a combination of at least two detrimental mechanisms: 1) direct action on liver vascular endothelium by inhibition of prostacyclin biosynthesis; 2) damage to the intestinal mucosa, followed by inflammatory and pro-coagulant activation of portal endothelium upon exposure to bacterial endotoxins.

**Conclusions:**

This case can be of interest to physicians, who should exert caution when prescribing NSAIDs for inflammatory pain in patients with background inflammatory dysfunctions of the portal vein endothelium.

## Background

Portal vein thrombosis (PVT) is a thrombotic obstruction of extrahepatic/intrahepatic portal venous system, associated with local and systemic risk factors [1,2]. Local factors include: liver cirrhosis; hepatobiliary and pancreatic malignancy; abdominal inflammation, infections and surgery. Systemic risk factors encompass congenital and acquired conditions. Congenital genotypes comprise: mutations leading to antithrombin, protein C and protein S deficiency, with loss of anticoagulant activity; mutations of prothrombin and factor V Leiden genes, with gain of procoagulant activity. Other inherited risk factors include high levels of factors VIII, IX and XI, and hyperhomocysteinemia. Acquired risk factors are: myeloproliferative disorders; anti-phospholipid syndrome; chronic inflammatory diseases; obesity; oral contraceptive intake; pregnancy and post-partum period [2-6].

Signs of acute PVT include: intestinal congestion, with abdominal distension and pain; splenomegaly; ascites; diarrhea; rectal bleeding; nausea and vomiting; anorexia; fever; lactacidosis; sepsis. Chronic PVT can be asymptomatic and characterized by portal cavernoma and portal hypertension, cholestasis, splenomegaly, ascites, gastro-esophageal varices, and pancytopenia [3].

This report describes a case of acute PVT in a hepatitis C virus (HCV)-positive elderly patient treated with indomethacin for acute back pain.

## Case presentation

On May 2010, a 79-year-old man was hospitalized for severe postprandial abdominal pain, nausea and vomiting. His medical history revealed a HCV-related mild non-progressive chronic hepatitis (diagnosed in 1985; Child**-**Pugh class A), never treated with anti-HCV therapy. He had a normal body weight (BMI: 25), and did not present any other risk factor for thrombosis, such as smoking, alcohol intake or diabetes mellitus. Furthermore, his family history was negative for bleeding or clotting disorders. Since 1996, he was under treatment with lisinopril/hydrochlorothiazide (20/12.5 mg/day) for hypertension. Fifteen days prior hospitalization, he was placed under treatment with indomethacin (100 mg/day) for back pain. After 11 days of indomethacin therapy, the patient started to complain of dyspepsia and abdominal pain, which worsened rapidly leading to his hospitalization.

At admission, physical examination revealed a tractable and painful abdomen. The patient reported a past sporadic use of anti-inflammatory non-steroidal drugs (NSAIDs) and no previous indomethacin intake. Blood tests showed increased neutrophil count and alanine aminotransferase, as well as moderate reductions of platelet count, activated partial thromboplastin time (aPTT) and antithrombin III. However, the prothrombin fragment F1+2 was not assessed, and therefore a clear information about the status of thrombin generation in this patient could not be obtained. Notably, all these parameters were within their normal ranges on a previous assessment, performed about 2 months earlier (i.e. 45 days before starting indomethacin), during a scheduled visit for monitoring his liver function and general health condition. Relevant laboratory parameters, recorded before and throughout the hospitalization, are summarized in Table 1. Abdominal ultrasonography displayed: liver with regular margins and fine heterogeneous structure; ascites involving all quadrants; increase in the diameter of portal vein (17 mm). Both ascites and portal vein ectasia had not been detected on recent past follow-up ultrasounds. Based on leukocytosis, the clinicians hypothesized a bacterial infection, and started a treatment with ciprofloxacin (1000 mg/day). In addition, indomethacin was discontinued, as its possible involvement in the pathogenesis of abdominal symptoms was suspected.

**Table 1:** Time course of laboratory parameters

**Parameters (reference range)**	**2 months before admission**	**Admission**	**Day 3**	**Day 6**	**Day 7**	**Day 8**	**Day 11**	**Day 13**
**Red Blood Cell (4.5-6,00 10**^**6**^**/μl)**	4.82	5.53	4.70	4.46	-	4.17	4.15	-
**White Blood Cell (4.5-10,00 10**^**3**^**/μl)**	9.15	**18.94**	**15.49**	**11.26**	-	9.16	8.37	-
**Neutrophil (1.8-7.5 10**^**3**^**/μl)**	4.21	**16.36**	**11.29**	**7.51**	-	5.70	4.37	-
**Platelet (150–400 10**^**3**^**/μl)**	322	**121**	**126**	183	-	255	367	-
**AST (<40 U/l)**	40	40	33	**85**	-	**69**	36	-
**ALT (<41 U/l)**	**68**	**74**	**53**	**131**	-	**108**	**61**	-
**GGT (<60 U/l)**	35	36	24	35	-	-	-	-
**Total Bilirubin (<1.2 mEq/l)**	1.11	1.15	**1.21**	-	-	-	-	0.22
**Lactate dehydrogenase (135–225 U/l)**	-	211	**244**	**240**	-	-	-	-
**Prothrombin activity (80-130%)**	91	97	92	84	-	91	92	-
**INR (0.87-1.12)**	1.08	1.02	1.05	1.10	-	1.06	1.05	-
**aPTT (25.1-36.5 sec)**	30.9	**23.0**	**22.8**	26.7	26.1	25.5	30.7	-
**aPTT Ratio (0.84-1.22)**	1.01	**0.77**	**0.76**	0.89	0.87	0.85	1.02	-
**Fibrinogen (200–450 mg/dl)**	347	344	404	-	329	367	333	-
**Antithrombin (83-128%)**	95	**67**	-	**68**	98	112	**72**	96
**D-dimer (< 0.3 mg/dl)**	-	-	-	**5.74**	**3.29**	**2.86**	**1.23**	**1.07**
**C-Reactive Protein (< 0.500 mg/dl)**	-	-	**13.700**	-	-	-	-	-
**Albumin (3.6-4.9 g/dl)**	3.7	-	**2.8**	-	-	-	-	**3.3**

Abbreviations: *AST* aspartate aminotransferase; *ALT* alanine aminotransferase; *GGT* gamma glutamyltransferase; *INR* international normalized ratio; *aPTT* activated partial thromboplastin time; *aPTT Ratio* activated partial thromboplastin ratio.

On day 4, a follow-up abdominal ultrasound confirmed previous findings and displayed diffuse small bowel distension, without colonic involvement. An upper digestive endoscopy did not reveal erosions, ulcerations, mucosal dysplasia or gastro-esophageal varices. On day 5, a virological blood screening was performed: cytomegalovirus (CMV) IgM index was negative, while IgG levels were positive, suggesting a past infection; anti-HCV antibody, anti-HCV IgM and HCV core antigen were positive; screening tests for hepatitis B virus were negative. A CT-scan of chest and abdomen, with intravenous contrast medium, revealed: massive thrombosis of portal trunk; partial thrombosis of left portal vein branch and superior mesenteric vein; mild ascites and ileal distension, with parietal thickening; mild lymphadenopathies in the hepatic hilum, celiac trunk and intercavo-aortic region; and no sign of cancer or suspected lesions. A diagnosis of PVT was then made, and a treatment with fondaparinux (7.5 mg/day) and antithrombin infusion (2000 UI) was started.

On day 7, a decrease in protein C (30%, reference range 72-146%) and protein S (30%, reference range 70-140%) was detected. However, both parameters had never been altered in past assessments. A genetic screening did not show relevant mutations. Treatments with intravenous antithrombin and oral ciprofloxacin were discontinued. On day 11, a follow-up abdominal CT scan, with contrast medium, displayed the persistence of thrombosis in portal trunk, left portal vein branch and superior mesenteric vein, with a reduction of intestinal distension and ascites. Moreover, an abdominal Doppler confirmed an almost complete PVT. Throughout the hospitalization, the patient had some episodes of red bloody stool excretion, which were ascribed to his venous stasis.

On day 15, the severity of postprandial pain decreased significantly, and the patient was discharged. After discharge, he remained on fondaparinux for 12 days, and then he was switched to warfarin with the goal of maintaining the international normalized ratio within the therapeutic range of 2–3. Thirty days later, a follow-up ultrasound did not show appreciable signs of PVT. At 2 years from discharge, several follow-up visits have shown that the patient persists on a stable mild liver disea*s*e and he has not experienced further thrombotic events.

## Discussion

In the present case, the time interval elapsing between the start of indomethacin treatment and the event onset suggests a role of this NSAID as the triggering agent of PVT. According to the available medical information, in this patient a prothrombotic condition has developed during the 2 months ranging from the last blood analysis, performed before his admission, and the onset of the abdominal symptoms leading to his hospitalization. Therefore, the possibility that his mild chronic hepatitis might have contributed to the occurrence of PVT cannot be ruled out, even though his clinical history and his previous records of coagulation parameters give a weak support to the hypothesis that the underlying liver disease played a predominant pathogenic role in the development of PVT. In this context, when considering the possible association of indomethacin with PVT, two possible scenarios can be proposed: 1) the patient had a subclinical prothrombotic state, and indomethacin acted as the triggering agent of an overt PVT; 2) indomethacin elicited primarily a prothrombotic state, which subsequently evolved towards PVT in the presence of a susceptible portal endothelium. Whatever the pathogenic picture, it is important to consider that the patient recovered promptly from PVT after indomethacin dechallenge and the start of adequate anticoagulant therapy. Moreover, the patient has not experienced any further thrombotic event after about a two-year follow-up from his discharge. A causality assessment of the association of indomethacin therapy with the occurrence of PVT, using the Naranjo probability scale, has scored as possible [7].

In patients with severe chronic liver diseases, such as cirrhosis, PVT can occur as a complication [8]. However, our patient had a mild HCV-related chronic hepatitis without signs of significant progression, as documented by previous liver ultrasound patterns and lack of portal hypertension and gastro-esophageal varices. Acute viral infections, including CMV, represent other possible risk factors for PVT, but, in our case, serologic evaluations were consistent with a past CMV infection [9]. Other systemic risk factors, including congenital defects, do not appear to have played a relevant role in the occurrence of the present PVT event. Moreover, since the levels of antithrombin III, platelet count, aPTT, protein C and protein S were not altered in close proximity of PVT onset, as shown by previous follow-up assessments (Table 1), these deficiencies were likely a consequence of PVT, rather than acting as contributing factors. In support of this contention, based on information from current literature [10], the slight alterations of coagulation parameters, recorded at admission, can be explained by their consumption in the acute phase of thrombosis and the reduced hepatic blood flow consequent to PVT. In this light, the hypothesis of indomethacin as the triggering agent of PVT would be strengthened further.

Besides a background chronic anti-hypertensive therapy, the patient had been recently started on treatment with indomethacin at the time of his symptom presentation. We therefore performed a MEDLINE search on these drug treatments until December 2011. Key search terms were: indomethacin, non-steroidal anti-inflammatory drugs, NSAIDs, lisinopril, angiotensin-converting enzyme inhibitors, ACE-Is, hydrochlorothiazide, diuretics, portal vein thrombosis, venous thrombosis and thromboembolism. According to our literature search, cases of indomethacin-related PVT have not been previously published. Nevertheless, we were able of retrieving 4 unpublished cases of PVT in patients taking indomethacin, by exploring the US Food and Drug Administration and user community through the eHealthMe searching system [11]. Moreover, a case–control study, on the association between the use of NSAIDs, including indomethacin, and the risk of venous thromboembolism, reported an overall incidence rate ratio of 2.51 (95% confidence interval [CI] 2.29-2.76). Of note, this study showed also that in new users (first-ever exposure to NSAIDs within 60 days before thrombotic event) the risk increased to 4.56 (95% CI 3.85-5.40) [12]. In addition, another case–control study highlighted an increased risk of symptomatic pulmonary embolism in patients taking NSAIDs (odds ratio: 2.39, 95% CI 2.06-2.77). In this analysis, the risk was higher for traditional non-selective NSAIDs over non-users and the overall risk for NSAIDs was higher in the first 30 days of exposure (odds ratio: 4.77, 95% CI 3.92-5.81), as compared with chronic (<1 year; odds ratio: 1.83, 95% CI 1.47-2.28) or long-term use (>1 year; odds ratio: 2.14, 95% CI 1.48-3.09). This association can be partly explained by the underlying medical conditions, which may hamper the haemostatic balance [13]. For instance, the intake of drugs inhibiting prostaglandin G_2_ synthase, such as acetaminophen, during pregnancy has been associated with an increased risk of preeclampsia and thromboembolic events, and this effect could be the consequence of a reduced prostacyclin production during childbearing [14].

**Figure 1:**
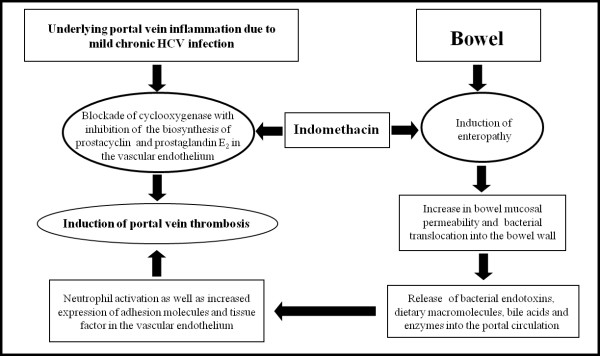
Proposed mechanisms accounting for the pro-thrombotic action of indomethacin in the present case of portal vein thrombosis

The present patient was a new indomethacin user, and he disclosed a history of sporadic NSAID intake. In this regard, at least two mechanisms might be advocated to support a role of indomethacin as the triggering agent of PVT (Figure 1). The first mechanism calls into play a direct action of indomethacin on liver vascular endothelium. Nygård et al. have demonstrated that the exposure of cultured human umbilical vein cells (HUVEC) to indomethacin, either alone or in the presence of lipopolysaccharide (LPS), led to an increase in procoagulant activity [15]. In particular, LPS application to endothelial cells resulted both in the induction of procoagulant activity and the release of 6-keto-prostaglandin F_1α_ (the stable metabolite of prostacyclin) and prostaglandin E_2_, as protective responses deputed to maintain the patency of vascular blood flow. When HUVEC were cultured in the presence of indomethacin and LPS, the prostanoid production was suppressed, and the procoagulant response increased up to the induction of pro-thrombotic alterations [15]. In keeping with these findings, a study on rats with portal hypertension showed that the concomitant administration of indomethacin and low-dose aspirin resulted in a loss of antithrombotic protection by aspirin on portal endothelium [16]. Based on this knowledge, it is conceivable that in the present patient, who had a background of portal endothelial inflammation maintained by chronic HCV infection [17], indomethacin did precipitate PVT by blocking the endothelial biosynthesis of prostacyclin (Figure 1). A second mechanism can be related to the expected indomethacin-induced intestinal mucosal damage, with consequent enterobacterial translocation into the bowel wall and release of bacterial toxins into the portal circulation [18,19]. Indeed, most of NSAIDs, including indomethacin, are endowed with acidic/lipophilic chemical structures, which allow them to elicit intestinal injuries, even in the absence of upper digestive lesions, mainly by means of topical damaging mechanisms [19,20]. As a consequence, the abnormal increase in bowel mucosal permeability allows bacteria, together with other toxic agents (e.g., dietary macromolecules, bile acids, enzymes of pancreatic juice), to penetrate into the enteric tissues and activate inflammatory cascades [19,21]. In particular, Gram- bacterial endotoxins, such as LPS, are potent inflammatory stimuli of vascular endothelium, where they can induce the expression of adhesion molecules and tissue factor (TF), interleukin-1 production, and stimulation of arachidonic acid metabolism (Figure 1). TF acts as a cell surface receptor for the circulating serine protease factor VIIa, and the formation of TF/factor VIIa complex results in the activation of blood coagulation through the extrinsic pathway [17]. In addition, the older age, as in this case, has been recognized as a specific risk factor for the development of bowel injury in patients taking NSAIDs. Indeed, a pooled analysis of three randomized studies, performed on 34,701 elderly patients treated with etoricoxib or diclofenac for osteoarthritis/rheumatoid arthritis, demonstrated an hazard ratio of 1.98 (95% CI 1.45-2.71) for the occurrence of intestinal damage in patients aged over 65 years [22]. Of note, a mucosal damage, elicited by indomethacin at the intestinal level, may have contributed to: 1) the neutrophil activation and high levels of C-reactive protein, which were recorded at the patient admission; 2) the episodic excretion of bloody stools, as observed in this patient during his hospitalization [23]. Finally, it is noteworthy that close correlations between inflammatory bowel diseases/enteropathies and prothrombotic conditions, such as PVT, have been well documented in the medical literature [2,3,24,25]. 

## Conclusions

The present report suggests that in patients with background inflammatory conditions of portal vein endothelium, indomethacin may precipitate thrombotic events, likely through the inhibition of endothelial prostacyclin biosynthesis and alterations of intestinal permeability. Moreover, in these patients the older age may represent an additional risk factor for NSAID related bowel damage, thereby further increasing the risk of endothelial activation and PVT. Accordingly, in these patients the option of prescribing non-NSAID analgesics might be considered when pain management is the main issue, while caution should be exerted when NSAID prescription is deemed to be unavoidable.

## Consent

Written informed consent was obtained from the patient for publication of this case report. A copy of the written consent is available for review by the Editor-in-Chief of this journal.

## Abbreviations

NSAIDs: Non-steroidal anti-inflammatory drugs; PVT: Portal vein thrombosis; HCV: Hepatitis C virus; CMV: Cytomegalovirus; CT: Computed tomography; ACE-Is: Angiotensin-converting enzyme inhibitors; HUVEC: Human umbilical vein cells; LPS: Lipopolysaccharide; TF: Tissue factor.

## Competing interests

The authors declare that they have no competing interests.

## Authors’ contributions

All authors contributed in a significant way in the steps of processing the patient history as well as writing and editing the manuscript. SMa performed: assessment of the case; literature review; writing of the manuscript. MT participated in the assessment of the case and helped to draft the manuscript. GP conceived the idea of reporting this case and participated in the assessment of the case. ST and SMo contributed to the assessment of the case and literature review. The senior authors FM and CB provided geriatric and pharmacological expertise, and carried out the internal review of the manuscript. All authors read and approved the final version of the manuscript.

## Pre-publication history

The pre-publication history for this paper can be accessed here:

http://www.biomedcentral.com/1471-2318/12/69/prepub
